# Ear tissue as a diagnostic sample for pestivirus detection in semi-domesticated Eurasian tundra reindeer (*Rangifer tarandus tarandus*) in Norway

**DOI:** 10.3389/fmicb.2025.1688206

**Published:** 2025-11-20

**Authors:** Ester Malmström, Morten Tryland, Thomas Passler, Alice Becker, Stine Bull-Aurbakken, Scott Silvis, Rachel Phillips, Shollie Falkenberg

**Affiliations:** 1 Department of Forestry and Wildlife Management, University of Inland Norway, Koppang, Norway; 2 Department of Clinical Sciences, Auburn University College of Veterinary Medicine, Auburn, AL, United States; 3 Animal Health Research, Auburn University College of Veterinary Medicine, Auburn, AL, United States; 4 Department of Pathobiology, Auburn University College of Veterinary Medicine, Auburn, AL, United States

**Keywords:** persistent infection, bovine viral diarrhea virus, border disease virus, ear notch, polymerase chain reaction, antigen capture ELISA

## Abstract

**Introduction:**

Although eradication programs have successfully controlled pestivirus infections in domestic livestock across Fennoscandia, serological evidence suggests that several free-ranging, semi-domesticated reindeer herds are exposed to and possibly endemically infected with pestivirus(es). While the significant economic impact of pestiviruses on domestic animals is well documented, their effects on reindeer remain poorly understood. Attempts to isolate and characterize these pestiviruses from seropositive reindeer herds have so far been unsuccessful, despite analyses of serum and nasal swab samples by multiple studies. Ear tissue is commonly used to detect cattle persistently infected (PI) with pestivirus and utilized for both screening and controlling infection. Despite its practicality in cattle, ear tissue has not been utilized for the demonstration of pestivirus in reindeer. The current study aimed to examine ear tissue as sample material for the detection and isolation of pestivirus in Norwegian semi-domesticated reindeer herds.

**Methods:**

Ear tissue from 3,453 reindeer calves from three geographically distinct locations were assessed by conventional reverse transcriptase polymerase chain reaction (RT-PCR), antigen capture ELISA (ACE), and virus isolation.

**Results:**

A total of 24 (0.7%) individual ear tissue samples were considered potentially positive by RT-PCR but were negative by ACE, and no virus could be isolated from any of the samples. Three commercially available reverse transcription quantitative polymerase chain reaction (RT-qPCR) assays for the diagnosis of bovine viral diarrhea virus (BVDV) were also employed from which a CT-value of less than 40 was detected in only one sample (CT 36.95).

**Discussion:**

While potential positive ear tissue samples were observed in this study, it is unknown if low viral load, pestivirus genetic diversity, or sample suitability contributed to the inability to confirm pestivirus-specific RNA nor viable virus particles in the samples. The impact of pestivirus infections on health and welfare of reindeer and effect on eradication programs in Fennoscandian livestock remain undetermined and the results from this study emphasize the critical need for multidisciplinary research regarding this topic.

## 1 Introduction

Reindeer husbandry generates a livelihood for many people in Fennoscandia (in this context defined as Sweden, Norway and Finland) and has an equally significant cultural value, especially for the indigenous Sami people. Reindeer meat and other products are sold as delicacies in grocery stores, which are supplied by 16 registered reindeer slaughterhouses in Norway (Landbruksdirektoratet, 2024a). Recent assessments estimate that 70,650 Norwegian reindeer were slaughtered during the year 2023–2024, which generated 1,626 tons of meat for human consumption (Landbruksdirektoratet, 2024b).

In contrast to typical farming of domestic animals, Fennoscandian reindeer husbandry mainly utilizes natural grazing lands with free-ranging animals throughout the year, which contributes to mortality from predation and winter/spring starvation (Nieminen et al., 2013; Tryland, 2013; Mørk et al., 2024). Reindeer calves are born in remote locations and are only monitored/counted for during summer and/or fall round-ups, making it difficult to identify the exact causes of morbidity and mortality. Factors such as poor nutrition, stress, illness, and infectious diseases may contribute to decreased reindeer fertility and overall poor calf survival (Laaksonen, 2016). Additionally, co-grazing with free-ranging domestic (sheep and occasionally cattle) and wild ruminants occurs in Norway (Utaaker et al., 2023), which increases the potential for general disease transmission between species.

Pestiviruses are single-stranded, positive-sense RNA viruses that can infect several species in the mammalian order *Artiodactyla*. Among domestic species, infections are common in cattle, swine, small ruminants, and new world camelids (Brock, 1995; Aguirre et al., 2014; Crilly et al., 2018; Renson and Le Potier, 2022), but wild mammals such as pronghorn (*Antilocapra americana*) (Neill et al., 2014), and caribou (*Rangifer tarandus*) (Carlsson et al., 2019), are also susceptible. The pestivirus species infecting cattle and sheep are bovine viral diarrhea virus (BVDV) 1 & 2, and border disease virus (BDV), which were recently renamed as *Pestivirus bovis, Pestivirus tauri,* and *Pestivirus ovis*, respectively (Cox et al., 2025).

Despite the successful eradication of BVDV and BDV from domestic livestock in Fennoscandian countries (Hult and Lindberg, 2005; Løken and Nyberg, 2013; Autio et al., 2021), several studies over the last decades documented seropositive reindeer herds, including some with high seroprevalence rates. Finnish reindeer herds appeared to have lower seroprevalence rates of 0.7% (*N* = 596) (Tryland et al., 2023) and 2.5% (*N* = 122) (Omazic et al., 2019), compared to Norwegian herds, with 41.2% (*N* = 596) (Tryland et al., 2021) and 38% (*N* = 119) (Omazic et al., 2019), and Swedish herds with 49% (*N* = 132) (Omazic et al., 2019), and 32% (*N* = 1,158)(Kautto et al., 2012). Nevertheless, these studies collectively indicate that pestiviruses are endemic in Fennoscandian reindeer populations.

To date, uncertainty exists about whether the circulating pestiviruses resulted from spillover infections from domestic species prior to implementation of the eradication programs, or if reindeer harbor species-specific pestivirus(es) (Larska, 2015; Omazic et al., 2019). Recent serological studies in Fennoscandia suggested that pestivirus from seropositive animals are more antigenically related to BDV than BVDV (Kautto et al., 2012; das Neves et al., 2019), as is the case for the only pestivirus ever isolated from a captive reindeer (Reindeer-1 virus), which was housed in a German zoo (Becher et al., 1999).

Although pestiviruses are known to result in a variety of clinical manifestations (most importantly immunosuppression, reproductive losses, and persistently infected (PI) offspring), and contribute to significant economic losses to livestock production systems globally (Houe, 2003; Vilček and Nettleton, 2006; Evans et al., 2019), their long-term impact on many wildlife populations remains largely unknown (Vilček and Nettleton, 2006; Ridpath and Neill, 2016; Tryland et al., 2019). However, examples exist in which pestiviruses are associated with severe health outcomes in free-ranging wildlife, such as the population impacts of BDV in Pyrenean chamois (*Rupicapra pyrenaica pyrenaica*) in Spain (Marco et al., 2009; Serrano et al., 2015).

Ear tissue has become a preferred sample material for many BVDV eradication programs globally due to the ease of collection and reliability for viral detection in cattle (Zimmer et al., 2004; Presi and Heim, 2010; Graham et al., 2021). Another reason ear tissue is a preferred sample is the decreased risk of maternal antibodies intervening with the assay, which is likely when blood samples are analyzed (Zimmer et al., 2004). On the other hand, ear notches require diligence during sample processing and storage to preserve sample quality (Ridpath et al., 2009). The most commonly described detection assays for ear tissue are: immunohistochemistry (Brodersen, 2004; Bedeković et al., 2011), antigen capture ELISA (ACE) (Kuhne et al., 2005; Presi and Heim, 2010; Wernike and Beer, 2024), conventional reverse transcriptase polymerase chain reaction (RT-PCR) (Weinstock et al., 2001; Şevik, 2018; Monteiro et al., 2019), and quantitative reverse transcription polymerase chain reaction (RT-qPCR) (Presi and Heim, 2010; Dias et al., 2014; McDougall, 2021).

Despite successful demonstration and isolation of pestivirus from ear tissue in cattle (Kuhne et al., 2005; Bedeković et al., 2011), and wild ungulates (Pogranichniy et al., 2008; Passler et al., 2016; Wolff et al., 2016), ear notch tissues have not been reported for the demonstration of pestivirus in reindeer. Fennoscandian semi-domesticated reindeer herds routinely conduct annual ear markings of calves (i.e., manually fixating a calf and cutting a distinct owner-specific pattern into the ear cartilage) (Beach, 2007), ear tissue is through this tradition an easily accessible and convenient sample for research and diagnostic purposes. This provides an excellent opportunity to screen the majority of calves born into a herd in a given year.

The main objective of this study was to evaluate the suitability of reindeer ear tissue as a sample for detecting pestiviruses in this species. This study utilized a variety of methods for viral detection and characterization and aimed to generate experiences and data to aid in gaining further knowledge of pestivirus in reindeer populations.

## 2 Materials and methods

### 2.1 Animals, region, and ear tissue collection

This study was part of a comprehensive project investigating the effects of the increasingly common practice of supplementary feeding on reindeer welfare, behavior, health, and sustainability of reindeer herding. The animal use application for this project was granted by the Norwegian National Animal Research Authority (FOTS id 29948). Because the ear tissue collected resulted from annual calf marking activities by reindeer herders, no additional animal use approval was necessary to obtain the owner-collected tissue samples.

Approximately 2–4-months old reindeer calves were ear-marked as part of routine herding practices in July–September 2023. During this common cultural practice each reindeer owner applies their unique ear marking pattern, which is registered in the national earmark registry (Beach, 2007). The calves were manually caught by the reindeer owners, restrained on the ground, and then marked using a knife. This traditional marking procedure generated pieces of ear tissue, approximately 2 × 4 cm in size, which were collected for the purpose of this research project. The marking procedure was performed rapidly to minimize stress and injury, and calves were released immediately afterward.

**Figure 1:**
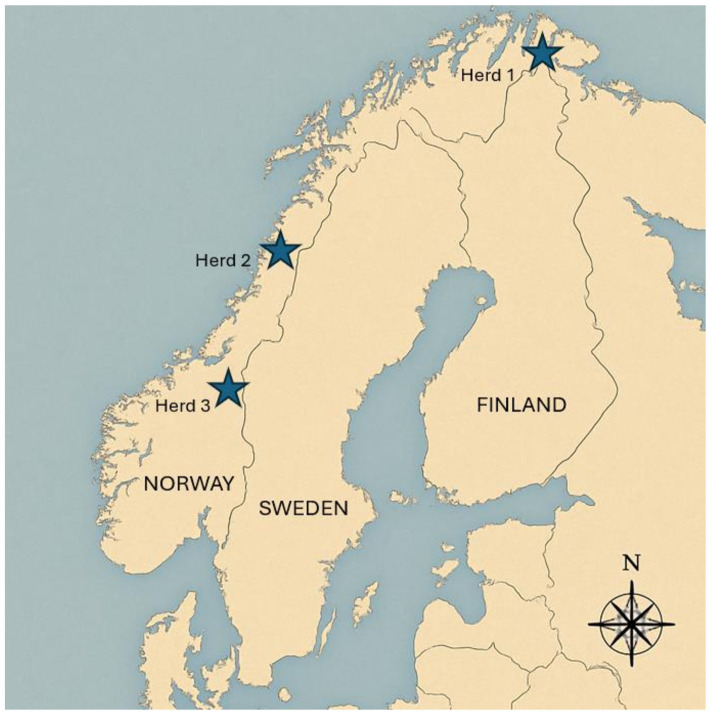
Map of Norway, Sweden, and Finland Showing the geographical locations of three Norwegian Reindeer Herds sampled for pestivirus analysis.

The calves belonged to three geographically distinct herds in Norway (Figure 1), and the number of calves sampled from each of the herds are presented in Table 1. Calf sex was not recorded. The pestivirus seroprevalence for two of the herds (Herd 1 and 3) had been investigated in 2013–2018, indicating that pestivirus was endemic (Herd 1 *N* = 14/99; 14%, Herd 3 *N* = 30/82; 37%), and the last herd (Herd 2) was chosen as it was neighboring another previously seropositive herd with (*N* = 57/110; 52%) from 2013 to 2018 (Tryland et al., 2021).

**Table 1:** Overview of positive and total counts for ear tissue pools (*n* = 10/pool) and Individual Ear Notch Samples Across Three Herds of semi-domesticated Eurasian tundra reindeer in Norway (2023).

Ear notch region	Total number of individual ear notches	Total number of pools	Number of positive pools	Number of potentially positive individual samples
Herd 1	2,080	208	10	18
Herd 2	50	5	0	0
Herd 3	1,320	132	2	6
Totals	3,453	345	12	24

Approximately 50–150 individual earpieces were collected daily and stored together in sample bags containing no more than 50 ear tissue samples per bag. After collection, the earpieces were placed into a refrigerator (4 °C), or a cooler containing ice packs for 2–8 h. At the end of each collection day, the ear tissue bags were further divided into pools of 10 earpieces and placed into smaller sample bags, temporarily stored in a −20 °C freezer for 1–2 weeks, and then transferred to a −80 °C freezer until shipping. At the end of the sampling period, a total of 345 pools with 10 individual tissue samples had been collected in each, representing 3,453 individual reindeer calves.

### 2.2 Cell lines and reference pestivirus isolates

**Figure 2:**
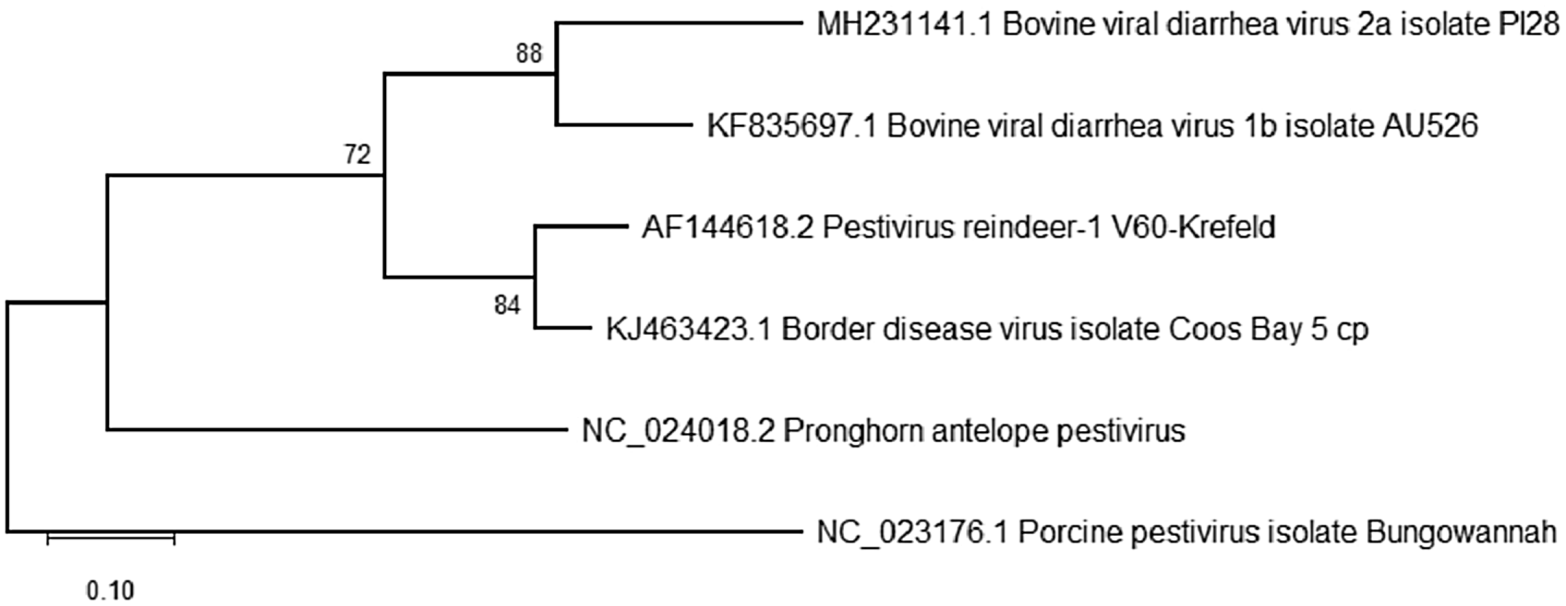
Phylogenetic analysis of the 5′-untranslated region (5’-UTR) for reference pestivirus isolates propagated and used as positive controls in the current study. Analyses were conducted in MEGA12 using the maximum likelihood model and bootstraps of 1,000 replicates. Accession numbers represent previously available full-length sequences and can be accessed at http://www.ncbi.nlm.nih.gov/pubmed/.

Five diverse pestivirus isolates that had previously been characterized including BVDV-1b (AU526; KF835697.1), BVDV-2a (PI28; MH231141.1), Reindeer-1 pestivirus (V60-Krefeld; AF144618.2), border disease virus (Coos Bay; KJ463423.1), and pronghorn antelope pestivirus (*Pestivirus antilocaprae*; NC_024018.2) were used to optimize viral propagation and detection procedures (Figure 2; Becher et al., 1999; Vilcek et al., 2005; Passler et al., 2014; Neill et al., 2019). Two primary cell lines, bovine turbinate (BTu) and ovine fetal turbinate (OFTu) cells were derived at the USDA-ARS-National Animal Disease Center, Ames, IA, United States, utilizing the previously reported methods described for harvesting and maintenance of bovine fetal testicle cells (Weber et al., 2017). Additionally, the immortalized Madin Darby Bovine Kidney (MDBK) cell line, previously obtained from the ATCC, was used to determine which cell line would be optimal for propagation of the genetically diverse pestivirus species. All cell lines were maintained in Minimal Essential Medium (MEM, Corning^®^, Glendale, AZ, United States), supplemented with 1% of antibiotic/antimycotic solution (A/A, Corning^®^, Glendale, AZ, United States) and 5% fetal bovine serum (FBS, Gibco/Thermo Fisher Scientific. Waltham, MA, United States). FBS was confirmed to be free of BVDV antigen and antibodies as previously described (Bauermann et al., 2014). Cells were also free from adventitious BVDV based on polymerase chain reaction (PCR) tests.

The BVDV-1b and BVDV-2a reference strains were viral stocks maintained in the laboratory, Reindeer-1 pestivirus isolate was received from University of Inland Norway, and border disease virus and Pronghorn pestivirus were received from the USDA-National Animal Disease Center Ames, IA. Propagation of the five reference viruses was accomplished by diluting 1 mL of each respective viral stock in 4 mL of MEM and inoculating a 175 cm^2^ flask of OFTu cells at approximately 60–70% confluency with the 5 mL of viral solution. After the addition of the viral inoculum, flasks were placed on a gentle rocker and incubated for approximately 1 h at 37 °C with 5% CO_2_. The viral inoculum was removed, and complete MEM media supplement was added to a final volume of 20 mL. Flasks were maintained at 37 °C with 5% CO_2_ in incubators for 96 h and monitored daily. After 96 h, flasks were frozen at −80 °C. After freezing, flasks were thawed, and contents were transferred into tubes for clarification by centrifugation at 800 × g for 10 min. The viral supernatant was poured into a new tube and passed through a 0.22 μm syringe filter and aliquoted into separated 1 mL aliquots for further use. The OFTu cell line was selected for virus isolation of samples and propagation given that superior viral titers were achieved for all five reference pestivirus isolates, when compared to BTu and MDBK cell lines in which Pronghorn pestivirus could not be propagated. Reference pestivirus isolates were used as positive control samples for sample extraction and conventional reverse transcriptase polymerase chain reaction (RT-PCR).

### 2.3 Processing of pooled ear tissue

The 345 bags with pooled earpiece tissue samples were sent on dry ice to another laboratory for further processing and stored there at −80 °C until analysis. The pools were further processed by excising a smaller sample from each individual earpiece sample while frozen using a commercial V-shaped ear notching tool (Agri-Pro ear notcher, Agri-Pro Enterprises-Iowa Inc., Iowa Falls, IA, United States), which provided a notch approximately 0.5 cm^2^ in size as is common when ear notches are collected from cattle for PI detection. The remaining earpiece tissues were left in the original pooled bags and stored at −80 °C. The 10 notches from each pool were placed into a 5 mL snap cap tube with 3 mL of cell culture medium (Gibco Opti-MEM^®^, Life Technologies Corporation, Grand Island, NY, United States) containing 1% of antibiotic/antimycotic solution (Corning^®^, Glendale, AZ, United States). This resulted in 342 tubes with 10 ear notches and 3 tubes with 11 ear notches in each. The tubes were vortexed for 10–15 s, submitted to two freeze–thaw cycles at −80 °C and room temperature, respectively, and centrifuged at 4000 × g for 4 min.

### 2.4 RNA extraction

One hundred and forty μL of supernatant from each pooled sample were used for RNA extraction. RNA extraction was performed using the QIAamp 96 Viral RNA Kit (Qiagen Inc., Valencia, CA, United States), according to the manufacturer’s recommendations.

### 2.5 Conventional reverse transcription-polymerase chain reaction

Extracted RNA from samples was assayed using a one-step RT-PCR based on the widely used primer set HCV90 (5’ CATGCCC ATAGTAGGAC 3′) and HCV368 (5’ CCATGTGCCATGTACAG 3′) (Ridpath et al., 1994; Vilcek et al., 1994; Ridpath and Bolin, 1998) targeting the 5′ untranslated region (5’ UTR) of the pestivirus genome and generating a 248-base pair (bp) amplicon. The reaction was performed with Promega (Promega Corporation, Madison, WI, United States) GoTaq Flexi system that consisted of 1X Green GoTaq Flexi buffer (Promega M891A), 4.5 mM MgCl_2_ (Promega A351H), 0.4 mM dNTPs (Promega C1141), 100 U M-MLV RT (Promega M170B), 1 U GoTaq DNA Polymerase (Promega M829B), 20 U RNasin Ribonuclease Inhibitor (Promega N251B), 0.1 μM of each primer, and nuclease-free water in a final volume of 25 μL.

RT-PCR was performed under the following conditions: RT at 50 °C for 1 h, initial denaturation at 95 °C for 4 min, 40 cycles of: 95 °C denaturation for 30 s, 50 °C annealing for 45 s and 72 °C elongation for 1 min; and a final elongation at 72 °C for 10 min, followed by a 4 °C hold. 5 μL of each PCR product was used for electrophoresis in a 1.5% agarose gel stained with GelRed^®^ (Biotium, Fremont, CA, United States), with band visualization under ultraviolet light. All samples yielding a visible band of appropriate amplicon size regardless of intensity were submitted for sequencing. Additionally, when a band that was approximately 250–300 bp in size was visualized, the remaining ear tissues comprising the pooled sample were further individually processed for virus isolation and extraction.

### 2.6 Nucleotide sequencing and phylogenetic analysis

RT-PCR products that yielded a visible band approximately 250–300 bp in size were submitted to Eurofins Genomics (Louisville, KY, United States) for sequencing. The PCR products were not cloned but sequenced directly in both directions. Quality samples yielded an amplification of a trimmed 248-bp sequence of the 5′ UTR. Any resulting quality sequences were edited and aligned using MAFFT version 7, and phylogenetic comparison among generated sequences was performed using the 12th version of the Molecular Evolutionary Genetics Analysis (*MEGA12*) software (Kumar et al., 2024), with Bungowannah virus (NC_023176.1) used as the outgroup. The evolutionary distances were inferred using the Maximum Likelihood analysis and Kimura 2 parameter + Invariant sites + Gamma distribution (K2 + G + I) as the best substitution model, and branch support estimated using bootstrap of 1,000 replicates (Figure 2).

### 2.7 Positive pool ear tissue—further processing

If a band was visualized for an ear tissue pool, another ear notch was collected as described above from each individual ear tissue contained in the pool. The individual ear sample was further processed by cutting it into small pieces, minced with scissors, followed by resuspension with 1 mL Opti-MEM and homogenization of the sample, then subjected to one freeze–thaw cycle. Samples were vortexed and 100 μL of supernatant from the tissue sample homogenate was used for viral isolation and 140 μL of sample supernatant for RNA extraction and RT-PCR, according to procedures as described above, aiming to identify the potential positive individual sample within each pool.

### 2.8 Viral isolation from individual ear tissue

Hundred μL of individual tissue supernatant as previously described were used to inoculate each respective 48-well plate that had been seeded for 24-h with OFTu cells, at a density of 2 × 10^5^ cells/mL. A positive control (Reindeer-1 pestivirus) and a negative (virus-free culture media) control were included on all plates. After the addition of the samples, plates were incubated for approximately 1.5 h at 37 °C with 5% CO_2_. The inoculum was removed and complete MEM media supplement as previously described (section 2.2) was added to a final volume of 500 μL. Plates were maintained at 37 °C with 5% CO_2_ in incubators for 96 h and monitored daily. Wells showing cell toxicity/cell death or bacterial/fungal contamination were recorded. After 96 h, plates were frozen at −80 °C until further passage. At each new passage, sample aliquots were obtained for RNA extraction and RT-PCR (sections 2.5 and 2.6) to assess for potential viral isolation, and samples were also used for further passage. Passaged material was used to inoculate new 48-well plates seeded with OFTu cells at a density of 2 × 10^5^ cells/mL for 24-h as described previously. Samples were subjected to a maximum of 6 passage attempts to isolate virus or obtain a sequence.

### 2.9 Commercial ACE and RT-qPCR

Commercially available pestivirus tests used for BVDV detection in cattle, including an ACE (ELISA BVDV PI x2 test; IDEXX Laboratories, Westbrook, ME, United States) targeting the E^rns^ protein of BVDV and three commercially available BVDV-detection quantitative RT-PCR (RT-qPCR) assays (Virotype^®^ BVDV RT-PCR; Indical Bioscience GmbH, Leipzig, Germany; VetMAX™ BVDV 4ALL; Thermo Scientific, Waltham, MA, United States; and RealPCR BVDV; IDEXX, Westbrook, ME, United States) were evaluated. The ACE and each of the three RT-qPCR were conducted according to the manufacturer’s recommendation and RNA for the RT-qPCR assays was extracted as previously described (section 2.4). Samples used in each assay included viral stocks for each of the reference pestivirus isolates (BVDV-1b, BVDV-2a, BDV, Pronghorn, and Reindeer-1; V60-Krefeld) that had previously been confirmed positive by conventional RT-PCR to assess the ability of the other assays to detect previously confirmed positive samples.

A subset of ear tissue samples, either those in which a band was visualized (*n* = 24) or those randomly chosen with no visible band (*n* = 24), were analyzed using ACE to further characterize samples previously identified as potentially positive or completely negative by conventional RT-PCR. The viral stocks from each reference pestivirus isolate were diluted (1:1) with supernatant from a negative reindeer ear notch to assess potential sample inhibition associated with the ear notch itself. In addition, 10-fold dilutions of each viral stock from 10^−1^ to 10^−6^ were made with PBS. RT-qPCR kits were also used to assess the subset of samples and the reference pestivirus samples.

## 3 Results

### 3.1 Cell line and assay validation using reference pestivirus isolates

**Figure 3:**
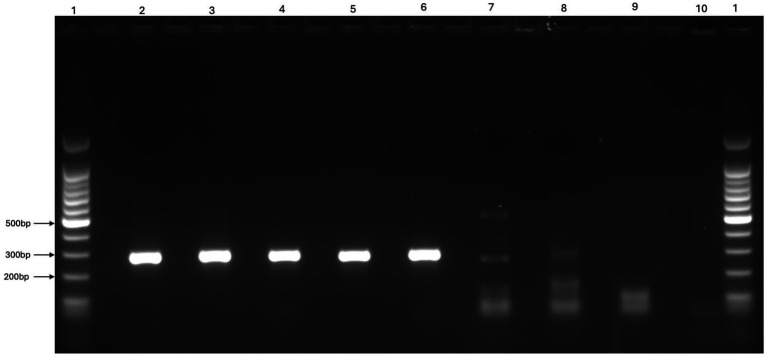
Representative gel image comparing reference *Pestivirus* strains (BVDV1b AU526, BVDV2a PI28, BDV Coos Bay, Pronghorn Virus, Reindeer-1 V60-Krefeld) propagated and used as positive controls in the current study with two potentially positive and one negative ear tissue sample “Lanes 1:1,000 bp ladders, Lane2: BVDV1b AU526, Lane 3: BVDV2a PI28, Lane 4: BDV Coos Bay, Lane 5: Pronghorn Virus, Lane 6: Reindeer-1 (V60-Krefeld), Lane 7: Strong band of ear tissue samples, Lane 8: Weak band of ear tissue samples, Lane 9: Negative band of ear tissue samples, Lane 10: Negative control”.

All five reference pestivirus isolates were successfully detected by conventional RT-PCR methods when propagated in the OFTu cell line. The pronghorn antelope isolate could not be detected by RT-PCR when propagated using the MDBK or BTu cell lines. Successful propagation and detection of each reference isolate was confirmed by visualization of a band of appropriate size under ultraviolent light (Figure 3) and subsequent sequencing of the PCR product associated with the band. Since the OFTu cell line supported the successful propagation and detection of all reference pestivirus strains, it was selected as the most suitable cell line for virus isolation from reindeer tissues.

### 3.2 Viral detection, isolation, and sequencing from ear tissue

A total of 345 pools were tested using RT-PCR, comprising samples from three regions: Herd 1 (208 pools), Herd 2 (5 pools), and Herd 3 (132 pools) (Table 1). A visible band consistent with a potentially positive sample was observed for 12 pools in total, 10 pools from Herd 1 and two pools from Herd 3 (Table 1). The individual samples from these 12 pools were consequently processed and analyzed individually. Bands were visualized from 24 individual samples, with 18 samples from Herd 1 and 6 samples from Herd 3. All samples generating visible bands, regardless of intensity, were submitted for sequencing and virus isolation. Additionally, all virus isolation passages were assessed by RT-PCR and any samples for which a visible band was observed were submitted for sequencing. A representative image of the band intensity and potential positive samples submitted for virus isolation and sequencing is shown in Figure 3.

Quality sequences were not successfully obtained from any pool or individual samples that were submitted. Similarly, virus was not successfully isolated from pooled or individual ear tissue samples as determined by lack of a visible band after passage and lack of quality sequences after 6 passage attempts. Following the 6th attempt, no band could be visualized.

### 3.3 Detection using commercial RT-qPCR

A subset of samples, totaling 48 individual ear tissue samples (24 samples for which a band had been visualized and 24 random samples without a band), in addition to 10-fold dilutions in PBS (10^−1^ and 10^−6^) and 1:1 dilution with negative ear tissue for the five reference pestivirus isolates were used to assess the three commercial RT-qPCR assays (Table 2). Only one sample (1/24) previously characterized as having a visible band was also positive by the Indical PCR assay (Ct 36.95). Although two samples (2/24) for which no band had previously been observed had Ct values of 38.56 and 37.93 by the IDEXX PCR. Otherwise, all other ear tissue samples were negative by RT-qPCR methods.

**Table 2:** Performance assessment of commercial pestivirus antigen capture ELISA (ACE) (IDEXX ELISA BVDV PI x2 test) and commercially available BVDV-detection quantitative RT-PCR assays; Indical Virotype BVDV RT-PCR, Thermo Scientific™ VetMAX™ BVDV 4ALL, and IDEXX RealPCR, in detecting pestivirus infection from reindeer ear tissue (notch).

Sample type	Samples detailed	Sample test results			
		IDEXX ACE	IDEXX PCR	Indical PCR	Thermo Scientific PCR
Ear notch	24 negative samples	0/24	2/24 (Ct 38.56 & 37.93)	0/24	0/24
	24 positive samples	0/24	0/24	1/24 (Ct 36.95)	0/24
Negative Ear notch + virus	BVDV1b (AU526)	pos	21.06	16.28	19.2
	BVDV2a (PI28)	pos	23.47	15.75	25.71
	BDV (Coos Bay)	pos	22.68	27.52	–
	Pronghorn	neg	–	–	–
	Reindeer-1 (V60)	pos	24.66	29.08	–
Virus	BVDV1b (AU526) stock	pos	17.87	14.97	13.81
	BVDV1b (AU526) 10^−1^	pos	21.48	20.3	18.84
	BVDV1b (AU526) 10^−2^	pos	25.28	24.68	24.26
	BVDV1b (AU526) 10^−3^	neg	29.14	28.48	28.87
	BVDV1b (AU526) 10^−4^	neg	33.21	32	32.23
	BVDV1b (AU526) 10^−5^	neg	35.76	34.62	35.13
	BVDV1b (AU526) 10^−6^	neg	40.32	36.65	–
	BVDV2a (PI28) stock	pos	18.87	15.09	17.92
	BVDV2a (PI28) 10^−1^	pos	24.02	19.21	23.75
	BVDV2a (PI28) 10^−2^	pos	27.04	23.69	29.64
	BVDV2a (PI28) 10^−3^	neg	30.83	28	33.58
	BVDV2a (PI28) 10^−4^	neg	34.13	31.52	35.62
	BVDV2a (PI28) 10^−5^	neg	36.42	34.94	–
	BVDV2a (PI28) 10^−6^	neg	42.43	–	–
	BDV (Coos Bay) stock	pos	20.03	25.43	36.53
	BDV (Coos Bay) 10^−1^	pos	24.63	30.3	–
	BDV (Coos Bay) 10^−2^	neg	30.04	34.24	–
	BDV (Coos Bay) 10^−3^	neg	34.37	37.16	–
	BDV (Coos Bay) 10^−4^	neg	36.91	36.01	–
	BDV (Coos Bay) 10^−5^	neg	–	–	–
	BDV (Coos Bay) 10^−6^	neg	–	–	–
	Pronghorn stock	neg	–	35.8	–
	Pronghorn 10^−1^	neg	–	–	–
	Pronghorn 10^−2^	neg	–	–	–
	Pronghorn 10^−3^	neg	–	–	–
	Pronghorn 10^−4^	neg	–	–	–
	Pronghorn 10^−5^	neg	–	–	–
	Pronghorn10^−6^	neg	–	–	–
	Reindeer-1 (V60) stock	pos	23.09	26.81	38.23
	Reindeer-1 (V60) 10^−1^	pos	29.15	32.4	–
	Reindeer-1 (V60) 10^−2^	neg	32.48	36.21	–
	Reindeer- 1(V60) 10^−3^	neg	36.53	–	–
	Reindeer-1 (V60) 10^−4^	neg	37.44	–	–
	Reindeer-1 (V60) 10^−5^	neg	40.02	–	–
	Reindeer-1 (V60) 10^−6^	neg	–	–	–

Given that all reference pestivirus isolates could be detected using conventional RT-PCR, the same viral stocks were used to assess the three RT-qPCR assays. All three RT-qPCR assays performed similarly when used to detect the BVDV-1b and BVDV-2a isolates, although the ThermoFisher assay tended to be less sensitive, and positives were not observed for the BVDV-1b 10^−6^, BVDV-2a 10^−5^ and 10^−6^ dilutions. The lack of sensitivity of the ThermoFisher assay was also evident as only the 10^−1^ dilution of both the BDV and Reindeer-1 isolates were positive, but no other dilutions were positive on this assay. In contrast, all BDV dilutions, up to the 10^−4^, were positive using the Indical and IDEXX assays.

Interestingly, Reindeer-1 positive samples were detectable by the IDEXX assay up to the 10^−5^ dilution but only up to the 10^−2^ dilution using the Indical assay. While viral titers may have varied for each viral stock, as observed by differences in Ct values, comparisons among each assay suggest varying sensitivity for each RT-qPCR assay.

None of the three RT-qPCR assays effectively detected the Pronghorn isolate. While the Indical assay detected Pronghorn pestivirus in undiluted viral stock (Ct 35.8), no other positives were observed for the dilutions. Additionally, no positives were observed for the viral stock or any other dilutions for the Pronghorn isolate when using the IDEXX or ThermoFisher RT-qPCR assays. Lack of sensitivity between assays is highlighted by the differing results for the BDV, Reindeer-1, and Pronghorn isolates.

### 3.4 Detection using commercial ACE

A duplicate sample from the subset of the 48 ear tissue samples, the dilutions in PBS (10^−1^ to 10^–6^), and 1:1 dilution with negative ear tissue for the five reference pestivirus isolates were used to assess the commercial ACE assay (Table 2). All 48 ear tissue samples were negative by the ACE.

Positive results were observed for the BVDV-1b and BVDV-2a dilutions from 10^−1^ to 10^–3,^ up to the 10^−2^ dilution for the BDV and the Reindeer-1 isolates. Similarly, when the five reference pestivirus isolates were diluted 1:1 with a negative ear tissue sample, a similar trend in detection was observed for the ACE. No positive ACE results were observed for any of the dilution series of the Pronghorn isolate (Table 2). The BVDV-1b, BVDV-2a, BDV, and Reindeer-1 isolates diluted 1:1 with negative ear tissue were positive by ACE, whereas the Pronghorn isolate diluted 1:1 with negative ear tissue was the only 1:1 dilution that was negative by ACE (Table 2).

## 4 Discussion

The apparently endemic pestivirus of Fennoscandian reindeer has been elusive and several unsuccessful attempts to isolate and characterize the virus have been carried out for some time (Kautto et al., 2012; das Neves et al., 2019; Omazic et al., 2019). Despite previous success in the demonstration and isolation of pestivirus from ear tissue of other wild ungulates (Pogranichniy et al., 2008; Passler et al., 2016; Wolff et al., 2016), ear notches (despite their easy accessibility and annual availability), have not been previously utilized for the detection of pestivirus in reindeer.

In this study, ear tissue from 24 reindeer calves (0.7%) yielded weak PCR bands of approximately 250–300 bp, which were initially considered potentially positive and subjected to further investigation. However, no virus was isolated, and no sequences could be obtained from either the original samples or subsequent passages. Furthermore, the observed bands did not match the exact size of the positive control reference strains and were most likely non-specific. Consequently, all initially suspected positive samples were ultimately classified as negative.

Although pooling of ear tissue is widely accepted for screening large numbers of domestic livestock samples and has shown to be a sensitive method (Driskell and Ridpath, 2006; Kennedy et al., 2006; Edmondson et al., 2007; Loy et al., 2025), it is possible that this approach might have reduced sensitivity of detection compared to analyzing individual ear notches in this study. Although 18 individually potentially positive samples originated from 10 different pools, indicating that positive samples were not confined to a limited number of pools (Herd 1), in Herd 3, six positive tissue samples were found in only two pools. Regardless, analyzing each sample individually from the start would have been preferable. However, due to the large sample size in this study, testing 3,453 samples individually was not pursued for financial and practical reasons.

To further investigate the sample material, we analyzed potentially positive and negative ear tissue with the commercial ACE kit. The results gave rise to concerns about the potential for low viral load in potentially positive ear tissue samples, which could cause difficulties when attempting demonstration and isolation of virus. Virus isolation of pestivirus typically relies on the presence of viable virus in tissues and tissue degradation can lead to false negative results (Ridpath et al., 2009). The low viral load in our samples could potentially be the result of handling and storage routines during field sampling, which could have contributed to a possible degradation of the virus.

Cattle owners are often encouraged to send ear tissue samples to laboratories using standard mail with ice packs, and it is possible that BVDV is either more stable or present at higher levels in bovine tissues compared to the pestivirus that appears to be endemic in semi-domesticated reindeer. One method of improving detection and isolation of virus from ear notches could be immediate transfer of samples into liquid nitrogen after sample collection and until a − 80° C freezer can be reached (Bedeković et al., 2011). While optimal storage environments could be utilized in most research studies, such conditions would be very impractical to achieve for reindeer herders during the annual calf marking, which in most cases take place in remote location with limited access to infrastructure.

Another potential reason for the failure to detect viral antigen in the ear tissue samples by the commercial BVDV ACE test could have been attributed to the presence of an antigenically divergent virus. While considered antigenically related, examples of divergent pestiviruses exist. For example, the E^rns^ protein of the pronghorn virus is divergent from the E^rns^ protein of BVDV (Vilcek et al., 2005; Neill et al., 2014; de Martin and Schweizer, 2022), and could not be detected by the commercial assays in our study, even when confirmed positive by other methods.

It is also possible that the composition of ear tissue from different hosts species affects the suitability of this sample type for pestivirus detection and could therefore be associated with decreased viral load. The reindeer ear tissues were noticeably thinner and covered in a much thicker coat of hair as compared to ear tissue collected from cattle. Thus, the most appropriate tissue for PI animal detection in reindeer and other free-ranging ungulates requires further assessment, similar to a study that investigated the BVDV antigen distribution in tissues from PI white-tailed deer tissue (Passler et al., 2012). Notably, the ACE successfully detected the reference pestivirus isolates (Reindeer-1, BDV, and BVDV 1 & 2) when combined with negative notch- suggesting that the reindeer ear tissue does not contain species-specific inhibitors, which theoretically could have contributed to the negative test results.

In species other than cattle, previous research demonstrated that ante mortem samples such as white blood cells and nasal swabs can contain high viral titers (Van Campen et al., 1997; Passler et al., 2007; Raizman et al., 2009; Passler et al., 2014; Peddireddi et al., 2018). Similarly, post mortem samples containing high viral titers included lymphoid tissue (Liebler-Tenorio et al., 2004; Deregt et al., 2005; Raizman et al., 2009; Crilly et al., 2018), aborted tissue/fetal remains (Lamm et al., 2009; Passler et al., 2014; Crilly et al., 2018) and/or the central nervous system (Fernandez et al., 1989; Montgomery et al., 2008; Passler et al., 2012). Another study demonstrated greatest BVDV antigen distribution in the hepatobiliary, integumentary, and reproductive organs of PI white-tailed deer, with the viral distribution varying from PI cattle (Passler et al., 2012), which indicates that pestiviruses may be distributed differently depending on viral properties, the host species, and host susceptibility and immunity (Liebler-Tenorio et al., 2004). Although other tissues have shown to harbor a higher viral load, this study utilized ear tissue due to its ease of access and its common use in diagnostic testing across other species. The inconclusive findings of this study suggest that ear tissue may not be an optimal sample type for pestivirus detection in reindeer, warranting further targeted investigation.

Identifying new pestiviruses presents several other challenges, including choosing the right viruses for comparative serological studies, selecting an appropriate cell line for virus isolation, and designing effective PCR primers (Vilček and Nettleton, 2006). The 5′-UTR is relatively conserved across pestivirus species and has served as a target region for the development of pan-pestivirus reactive primers (Vilcek et al., 1994; Vilček and Nettleton, 2006), and is commonly used in pestivirus genotype classification. The RT-PCR method and primers used for initial screening successfully detected a potentially positive sample targeting the 5’ UTR of the pestivirus genome, along with the selected reference pestivirus species (including the highly divergent Pronghorn strain) (Figures 2, 3).

Given the limited success with detection and subsequent sequencing, this study explored alternative methods for viral nucleotide detection, such as commercially available RT-qPCR. A recent study reported two cases in which novel pestiviruses were isolated from German cattle (Neill et al., 2019; Köster et al., 2024). In both cases, the whole-genome sequences showed the highest level of identity to strain Reindeer-1 pestivirus isolate. Both viruses yielded positive results in BVDV diagnostic test systems Pestivirus antigen detection in serum samples and ear notches using the commercially available BVDV Ag/Serum ELISA Plus Test (IDEXX, Liebefeld, Switzerland), as well as detection in various organs from both calves using the commercial BVDV RT-qPCR assay; Virotype BVDV 2.0 RT-PCR Kit” (Indical, Leipzig, Germany). The results indicated that cross-reactivity can be an important issue in pestivirus diagnostics, but also demonstrating that these methods can detect other, related pestiviruses (Köster et al., 2024).

None of the three RT-qPCR assays effectively detected the Pronghorn isolate in our study. Only the Indical assay detected Pronghorn pestivirus viral stock, with no other positives observed for any other dilutions or the IDEXX or ThermoFisher RT-qPCR assays (Table 2). In contrast, the conventional RT-PCR assay was superior and detected viral RNA from all the five reference pestivirus isolates (Figure 3). This discrepancy supports the conclusion that the methods employed in this study represented were highly sensitive for detecting even a potentially divergent pestivirus species in reindeer ear tissue, and that the difficulties in both demonstration and isolation of virus were not due to a highly divergent pestivirus, but rather a low viral load in the sampled tissue. It is possible that a reindeer-specific cell line may have improved viral recovery, but we used the OFTu cell line encouraged by its permissiveness to all five reference strains, suggesting it would also support replication of a potentially divergent reindeer pestivirus.

The only documented case of natural pestivirus infection in *Rangifer* spp., which was followed by successful isolation of the Reindeer-1 pestivirus, occurred in a captive reindeer at Duisburg Zoo in Germany in 1996 (Becher et al., 1999). This animal exhibited significant diarrhea and anorexia. Additionally, two reindeer experimentally inoculated with BVDV had various clinical signs such as bloody diarrhea, transient laminitis/coronitis, and nasal lesions (Morton et al., 1990). In contrast, other mammalian species, including Plains bison (*Bison bison bison*) (Deregt et al., 2005), llama (*Lama glama*) (Wentz et al., 2003), mule deer (*Odocoileus hemionus*) (Van Campen et al., 1997; Van Campen et al., 2001), and elk (Wapiti; *Cervus canadensis*) (Tessaro et al., 1999), did not have severe clinical signs when inoculated with BVDV. However, similar to cattle, white-tailed deer infected with BVDV may experience ill-thrift, death, fever, decrease in circulating lymphocytes, birth of persistently infected (PI) offspring, and reproductive losses as potential outcomes (Ridpath et al., 2007; Ridpath et al., 2008; Passler et al., 2016).

The significant impact of pestiviruses on the welfare of livestock and economic viability of farms worldwide is well described (Houe, 2003; Vilček and Nettleton, 2006; Evans et al., 2019). In contrast, the impact on wild and semi-domesticated reindeer populations remains poorly understood (Vilček and Nettleton, 2006; Larska, 2015; Tryland et al., 2019), which is also the case for numerous other cervid species (Ridpath and Neill, 2016). This is why the clinical impact of natural pestivirus infections in *Rangifer* spp. remains speculative, particularly at the population level and over the long term. However, based on similarity of clinical signs between livestock and wildlife hosts following natural and experimental infections, pestivirus infections of reindeer may possibly result in decreased milk production (Moerman et al., 1994; Arnaiz et al., 2021), lower body mass (Runyan et al., 2017), decreased immune response (Chase, 2013; Tao et al., 2013; Tesfaye Melkamsew et al., 2025), and decreased fertility (Barbudo et al., 2008; Burgstaller et al., 2016; Arnaiz et al., 2021).

It would be valuable to evaluate which role pestivirus infections in reindeer have in the findings of studies examining factors such as maternal and calf fitness, body mass (Ballesteros et al., 2013; Veiberg et al., 2017), and female milk production (Gjøstein et al., 2004). This is particularly important since restricted milk/feed intake can lead to nutritional deficiencies in calves, making them more susceptible to infectious diseases and likely reducing their chances of surviving the first winter (Ruong, 1982; Tryland, 2013).

The three herds investigated in our study had annual calf mortalities ranging from 20 to 50% during the time of our study (Landbruksdirektoratet, 2024b). The differences between areas in the number of calves after losses can mainly be attributed to factors such as climate, predation losses, or animal density and pasture quality and availability (Landbruksdirektoratet, 2024b). Fennoscandian reindeer are free-ranging, and their calves are born in remote areas with limited close monitoring during summer and fall round-ups. Therefore, uncertainty exists about causes of calf mortality spanning from the fall rut (conception) through the round-up season the following late fall/early winter, during which young calves are sorted to either be slaughtered or destined to be replacement stock (Landbruksdirektoratet, 2024b). As discussed above, uncertainty exists regarding the impact of pestiviruses present in Fennoscandian reindeer, and future research should evaluate the potential role pestivirus infections play in unexplained calf losses.

The Fennoscandian countries have declared freedom from BVDV infections in livestock due to successfully implemented eradication programs. By standards from ESA (the European Free Trade Association, Surveillance Authority) and the European Union (EU), Finland and Sweden have been officially considered free from BVDV since 2010 (Autio et al., 2021) and 2022, respectively, and only test at-risk cattle herds and perform random screenings of serum and milk. The Norwegian eradication program, which was started in 1992, successfully eliminated BVDV, with the last detection in cattle in 2006 (Løken and Nyberg, 2013). However, regular screenings for antibodies in bulk milk and blood are still conducted in the control program. Similarly, Norwegian sheep populations are considered BDV free today, with previously reported cases thought to be attributed to spillover from cattle (Mattilsynet, 2025). We agree with Köster et al. (2024), that determining the presence of pestiviruses in small and wild ruminant populations would provide valuable information for assessing risk factors, particularly in BVDV-free regions.

The influence of both apparent and endemic pestivirus on current eradication programs in Fennoscandia warrants extensive and detailed investigation. This should involve an interdisciplinary approach that connects epidemiological modeling, cervid ecology, veterinary practice, and pathological as well as microbiological investigations (Ridpath and Neill, 2016), especially as inter-species co-mingling is not uncommon (Utaaker et al., 2023). With this study, we aimed to be a part of this approach and contribute to closing the significant knowledge gap regarding pestivirus infections in reindeer.

## 5 Conclusion

Our goal was to assess whether easily accessible reindeer ear tissue could serve as a reliable sample for pestivirus detection, as it does in other species. Evaluation of these samples did not yield successful virus isolation or genetic sequences and ultimately led to a conclusion that the samples were negative. These results raise concerns about the reliability of using ear tissue as sample material for pestivirus detection in reindeer. It is unknown if low viral load, pestivirus genetic diversity, reindeer tissue, or non-specific bands led to the initial potential positive designation. The characteristics of the pestivirus endemic to reindeer, as well as its potential acute and long-term effects on reindeer health, herding economy and its influence on existing pestivirus eradication programs in livestock, remains unclear. This underlines the need to improve how samples are collected, to explore other tissue types that may be more suitable, and to refine future diagnostic techniques when investigating the endemic reindeer pestivirus.

## Data Availability

The raw data supporting the conclusions of this article will be made available by the authors, without undue reservation.
